# Quality assessment of cardiovascular magnetic resonance in the setting of the European CMR registry: description and validation of standardized criteria

**DOI:** 10.1186/1532-429X-15-55

**Published:** 2013-06-20

**Authors:** Vincenzo Klinke, Stefano Muzzarelli, Nathalie Lauriers, Didier Locca, Gabriella Vincenti, Pierre Monney, Christian Lu, Detlev Nothnagel, Guenter Pilz, Massimo Lombardi, Albert C van Rossum, Anja Wagner, Oliver Bruder, Heiko Mahrholdt, Juerg Schwitter

**Affiliations:** 1 Department of Cardiology, Center of Cardiac Magnetic Resonance (CRMC), University Hospital Lausanne, Lausanne, Switzerland; 2 Department of Cardiology, Fondazione Cardiocentro Ticino, Lugano, Switzerland; 3 Department of Radiology, University Hospital Lausanne, Lausanne, Switzerland; 4 Department of Cardiology, Klinikum Ludwigsburg, Ludwigsburg, Germany; 5 Department of Cardiology, Clinic Agatharied, Academic Teaching Hospital, University of Munich, Munich, Germany; 6 Clinical Physiology Institute / G. Monasterio Foundation, Pisa, Italy; 7 Department of Cardiology, VU Medical Centre, Amsterdam, The Netherlands; 8 Department of Cardiology, Hahnemann University Hospital, Drexel University, College of Medicine, Philadelphia, USA; 9 Department of Cardiology, Elisabeth Hospital Essen, Essen, Germany; 10 Department of Angiology, Elisabeth Hospital Essen, Essen, Germany; 11 Department of Cardiology, Robert Bosch Hospital Stuttgart, Stuttgart, Germany

**Keywords:** Cardiac Magnetic Resonance, Image Quality, Quality Score, Late Gadolinium Enhancement Images, Cine Images, Stress First Pass Myocardial Perfusion

## Abstract

**Background:**

Cardiovascular magnetic resonance (CMR) has become an important diagnostic imaging modality in cardiovascular medicine. However, insufficient image quality may compromise its diagnostic accuracy. We aimed to describe and validate standardized criteria to evaluate a) cine steady-state free precession (SSFP), b) late gadolinium enhancement (LGE), and c) stress first-pass perfusion images. These criteria will serve for quality assessment in the setting of the Euro-CMR registry.

**Methods:**

Thirty-five qualitative criteria were defined (scores 0–3) with lower scores indicating better image quality. In addition, quantitative parameters were measured yielding 2 additional quality criteria, i.e. signal-to-noise ratio (SNR) of non-infarcted myocardium (as a measure of correct signal nulling of healthy myocardium) for LGE and % signal increase during contrast medium first-pass for perfusion images. These qualitative and quantitative criteria were assessed in a total of 90 patients (60 patients scanned at our own institution at 1.5T (n=30) and 3T (n=30) and in 30 patients randomly chosen from the Euro-CMR registry examined at 1.5T). Analyses were performed by 2 SCMR level-3 experts, 1 trained study nurse, and 1 trained medical student.

**Results:**

The global quality score was 6.7±4.6 (n=90, mean of 4 observers, maximum possible score 64), range 6.4-6.9 (p=0.76 between observers). It ranged from 4.0-4.3 for 1.5T (p=0.96 between observers), from 5.9-6.9 for 3T (p=0.33 between observers), and from 8.6-10.3 for the Euro-CMR cases (p=0.40 between observers). The inter- (n=4) and intra-observer (n=2) agreement for the global quality score, i.e. the percentage of assignments to the same quality tertile ranged from 80% to 88% and from 90% to 98%, respectively. The agreement for the quantitative assessment for LGE images (scores 0–2 for SNR <2, 2–5, >5, respectively) ranged from 78-84% for the entire population, and 70-93% at 1.5T, 64-88% at 3T, and 72-90% for the Euro-CMR cases. The agreement for perfusion images (scores 0–2 for %SI increase >200%, 100%-200%,<100%, respectively) ranged from 81-91% for the entire population, and 76-100% at 1.5T, 67-96% at 3T, and 62-90% for the Euro-CMR registry cases. The intra-class correlation coefficient for the global quality score was 0.83.

**Conclusions:**

The described criteria for the assessment of CMR image quality are robust with a good inter- and intra-observer agreement. Further research is needed to define the impact of image quality on the diagnostic and prognostic yield of CMR studies.

## Background

Cardiovascular magnetic resonance (CMR) has become a robust and important diagnostic imaging modality in cardiovascular medicine. Its clinical utilization is growing rapidly [1,2] thanks to its ability to investigate several aspects such as cardiac morphology and function, myocardial tissue characteristics, and myocardial perfusion within a single diagnostic session. In addition, all this information is obtained safely [3] and at reasonable costs [4] without exposing the patient to potentially hazardous ionizing radiation [5]. However, CMR is a technically demanding investigation of a rapidly moving organ due to the mechanical heart action and motion related to breathing excursion. Thus, several factors may cause image artifacts or impaired image quality, which may finally result in reduced diagnostic accuracy [6]. Some artifacts are patient related while others are caused by insufficient care to technical aspects during the image acquisition, or by technical/physical limits of the imaging sequence itself. Even though much effort has been spent in technical developments aimed at optimizing the image quality, cardiac imagers involved in CMR are still, and quite frequently, faced with issues related to sub-optimal image quality. Considering the potential impact of image quality on diagnostic accuracy and patient management, objective criteria to evaluate image quality are needed. Different groups have pointed out the importance of improving quality measures for different cardiac imaging modalities [7,8]. However, to our knowledge, information on standardized quality criteria to assess CMR studies is scarce, unlike for other cardiac imaging modalities [9,10]. We therefore aimed to describe and validate well-defined standardized criteria to evaluate the quality of CMR studies performed at 1.5T and 3T, including the most frequently used sequences in clinical practice: i) cine steady-state free precession (SSFP) images, ii) late gadolinium enhancement (LGE) images, and iii) first-pass stress perfusion images. These criteria will serve for the assessment of the image quality in the setting of the Euro-CMR registry and its substudies [11].

## Methods

### Definition of the criteria to evaluate CMR image quality

**Figure 1:**
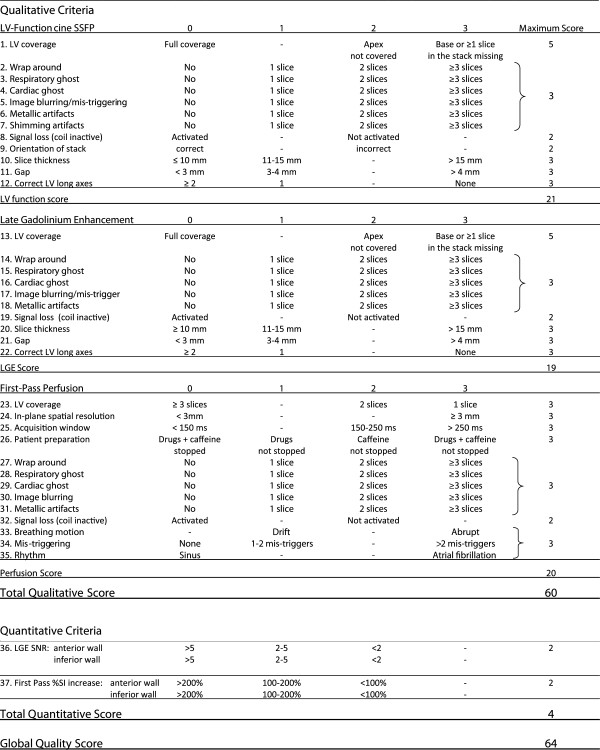
**Quality Evaluation of CMR Images.***Total qualitative score:* sum of qualitative scoring for SSFP images (12 criteria: range of scores 0–19, for LGE images (10 criteria: range of scores 0–19), and for perfusion images (13 criteria: range of scores 0–20). Total range: 0–60. *Quantitative LGE score:* 5 parameters measured yielding scores of 0–2 (SNR <2; 2–5; >5, respectively) for each the anterior and inferior LV wall. Total range of mean scores: 0–2. *Quantitative perfusion score:* 5 parameters measured yielding scores of 0–2 (%SI increase >200%; 100-200%; <100%, respectively) for each the anterior and inferior LV wall. Total range of mean scores: 0–2. *Total quantitative score:* sum of quantitative LGE and perfusion score. Range: 0–4. *Global quality score:* sum of total qualitative and total quantitative score: Range: 0–64.

Criteria to evaluate the image quality of i). cine SSFP images, ii). late gadolinium enhancement images, and iii). first-pass stress perfusion images were first defined *a priori* based on knowledge of the most common image artifacts, and on factors known to influence image quality. Specific criteria were defined for each type of imaging sequences (i, ii, iii) to obtain a numerical score that defines the image quality of the overall CMR study and of its modules (SSFP, LGE, stress perfusion). Thirty-five qualitative criteria were assessed by means of a scoring system with scores ranging from 0–3 for each criterion (higher scores meaning worse image quality, Figure 1). In addition, quantitative parameters were measured (see Figure 1) yielding signal-to-noise ratio (SNR) of normal myocardium for LGE images and % signal intensity (SI) increase during first-pass for the perfusion module, which were assessed in both, the anterior and inferior LV walls. Quantitative measures of SNR and %SI increase were scored 0–2 according SNR <2; 2–5; >5 and %SI increase >200%; 100-200%; <100%, respectively. A more detailed description of the quality criteria and scoring system is provided below and in Figure 1. Recommendations for SSFP, LGE, and first-pass perfusion acquisitions of adequate quality are given elsewhere [12].

### Cine SSFP CMR images

The image quality of cine SSFP images was evaluated based on 12 qualitative criteria (criteria 1–11 refer to the stack of short axis (SA) cine images).

The *coverage* (criterion 1 in Figure 1) of the left ventricle (LV) on the stack of SA cine images was the first quality criterion. A complete coverage from base to apex of the LV was required in order to guarantee accurate volume and functional measurements. The lack of the basal slice (=no atrial chamber visible in end-systole, hence no certainty that the base of the heart is covered completely) or lack of the apical slice (LV cavity still visible at end-systole) is in our experience the most frequent limitation regarding the coverage of the LV. Because the absence of the basal slice has an important impact on volume calculation, a higher score was given for the base versus apex. A mid-ventricular missing slice is resulting in a penalty as well. In order to balance the influence of this criterion in relation to other criteria, the maximum rating for this criterion is limited to 5 (no adequate basal [3 points] and apical [2 points] coverage even when ≥1 additional slice(s) missing). Regarding quality criteria 2 to 7 (wrap around, respiratory ghost, cardiac ghost, image blurring/mis-triggering, metallic artifacts, and shimming artifacts), 1 point was given if the artifact impeded the visualization of >^1^/_3_ of the LV endocardial border at end-systole and/or end-diastole on a single SA slice. If such artifact involved 2 slices or ≥3 slices, 2 and 3 points were given, respectively. In this study, the quality of RV visualization was not assessed.

**Figure 2:**
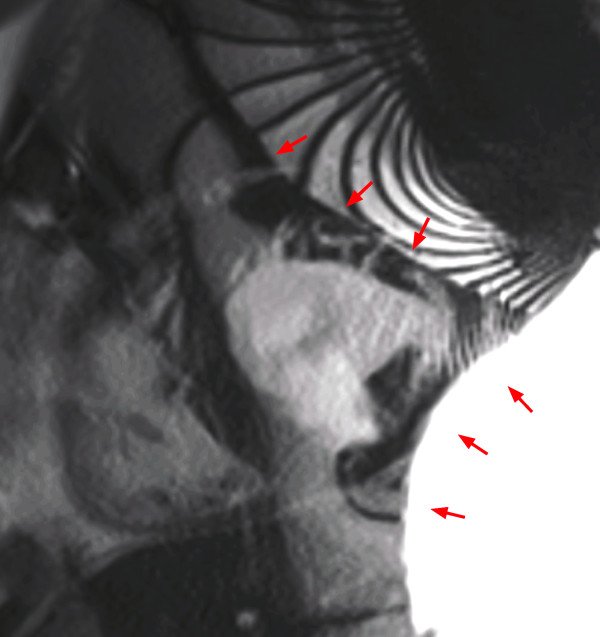
Wrap around in a cine SSFP sequence: Chest wall (located outside the field of view) is projecting onto the left ventricle (red arrows).

*Wrap around* artifacts (criterion 2) occur when the field of view is too small to cover the object to be scanned in the phase-encoding direction. It is easily recognizable as a portion of the object located outside of the field of view projecting into the image (Figure 2).

*Respiratory and cardiac ghosts* (criteria 3 and 4) are motion artifacts caused by respiratory or cardiac motion occurring during image acquisition, i.e. when a spin moves during the time of excitation. As a consequence, data sampling and reconstruction causes a mis-mapping of the signal. Such artifacts are typically projected onto the phase encoding direction on LGE images (for details, see below). Motion occurring during SSFP acquisitions generally causes image blurring. Nevertheless, the criterion of ghosts was added to cine SSFP acquisitions on Figure 1 to cover artifacts that may occur on future modifications of SSFP sequences.

**Figure 3:**
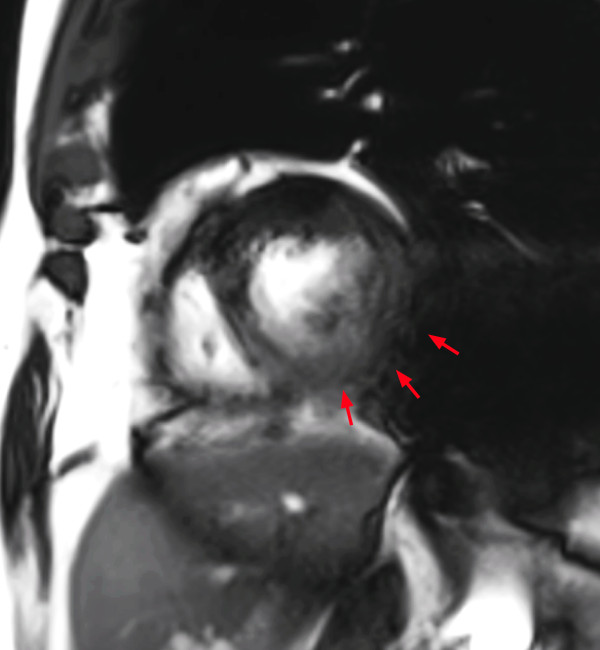
Image blurring/mis-triggering in cine a SSFP sequence: Respiratory motions, mis-triggering of the R-wave or irregular heartbeats induce a blurred aspect of the image (red arrows).

*Image blurring or mis-triggering* (criterion 5) is due either to irregular heartbeats such as extrasystoles or atrial fibrillation, to mis-triggering of the R-wave, or to respiratory motion. In case of segmented retrospectively gated acquisitions, which are considered the standard for cine imaging, signals used to reconstruct a specific phase of the cardiac cycle are collected during different phases of the cardiac cycle which in general results in image blurring of SSFP acquisitions (see Figure 3).

**Figure 4:**
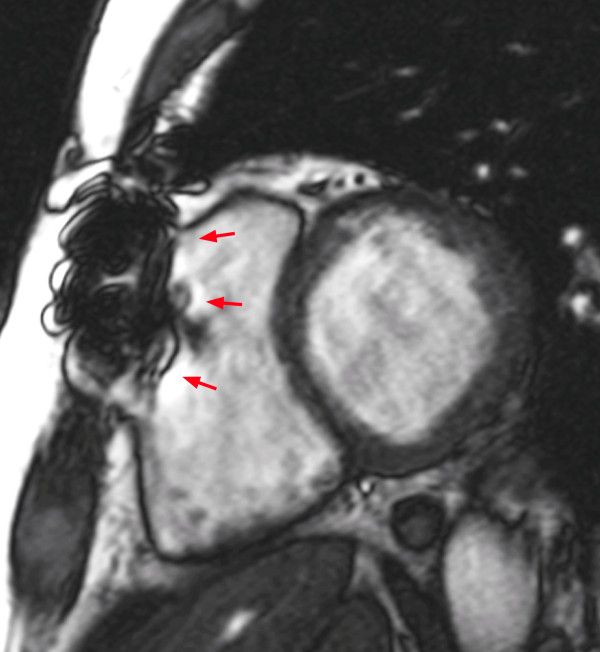
**Artifact in a cine SSFP sequence caused by ferromagnetic material: Sternotomy wires locally disturb the magnetic field (red arrows).** However, it is not considered a significant artifact in this case, since it does not extend onto the LV.

*Metal artifacts* (criterion 6) are due to the influence of metal (mainly iron) which deflects the magnetic field, thus changing the resonance frequency beyond the range, which is used for a given acquisition. As a consequence, the protons will not react appropriately to the excitation pulse and will therefore not be excited correctly causing a signal drop/distortion in the image (Figure 4).

**Figure 5:**
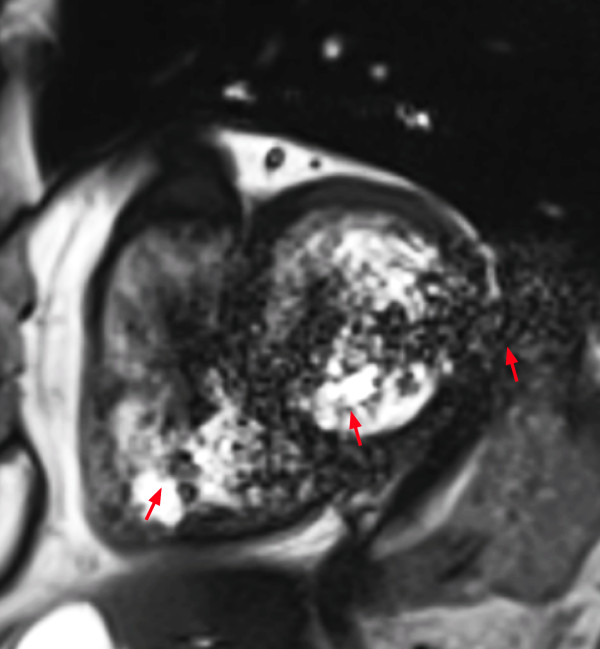
Shimming artifact in a cine SSFP sequence: Magnetic field inhomogeneities produce dark band and flow related (red arrows) artifacts on the LV.

Shimming artifacts (criterion 7) are due to inhomogeneity of the main magnetic field. SSFP acquisition schemes are particularly susceptible to such inhomogeneities of the magnetic field, that may cause banding artifacts (dark bands across the image caused by off-resonance) and/or flow related artifacts (Figure 5).

### Late gadolinium enhancement (LGE) CMR images

**Figure 6:**
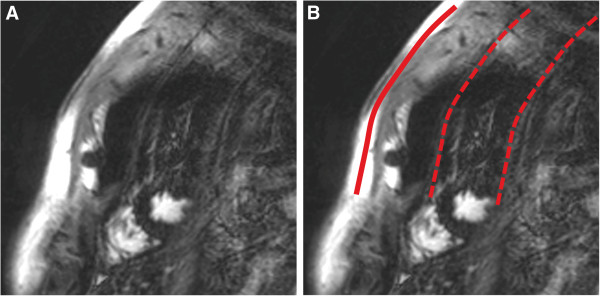
Respiratory ghost (indicated by red lines on the right image, B) in a LGE sequence: Respiratory motion during the image acquisition projects replicates of the chest wall onto the LV.

**Figure 7:**
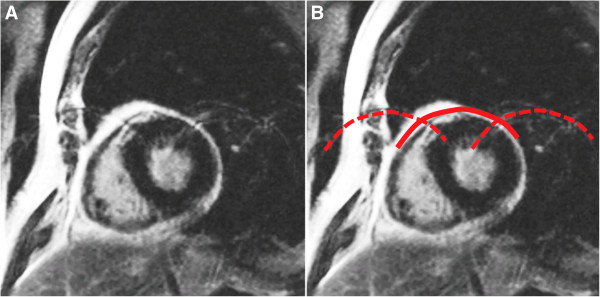
Cardiac ghost (indicated by red lines on the right image, B) in a LGE sequence: Cardiac motion during the acquisition is seen in this case as multiple replicates of LV contours in the phase-encoding direction.

The quality criteria applied to the LGE images included 10 qualitative criteria, which are quite similar to those used for the cine SSFP images (criteria 13–21 refer to the stack of SA images). Since a process leading to cardiac fibrosis or necrosis may be limited to a small region (i.e. in myocarditis), one point was given even if <^1^/_3_ of the myocardium was affected. In other words, one point was given if an artifact rendered a slice non-diagnostic, i.e. if e.g. a myocarditis could no longer be excluded/confirmed to be present in a given SA slice. Thus, for LGE the artifacts were read with higher sensitivity than for functional SSFP acquisitions. Full coverage (criterion 13 in Figure 1) of the LV is also required for an adequate diagnostic yield of LGE images. At the base and apex of the LV, the coverage is considered adequate if the position of the basal and apical LGE slice is the same as the basal and apical cine SSFP slice ±5 mm, respectively. Whereas respiratory motions often translate into blurred endocardial borders in cine SSFP sequences, they typically produce respiratory or cardiac ghosts in LGE sequences (criteria 15 and 16; see examples in Figures 6 and 7).

**Figure 8:**
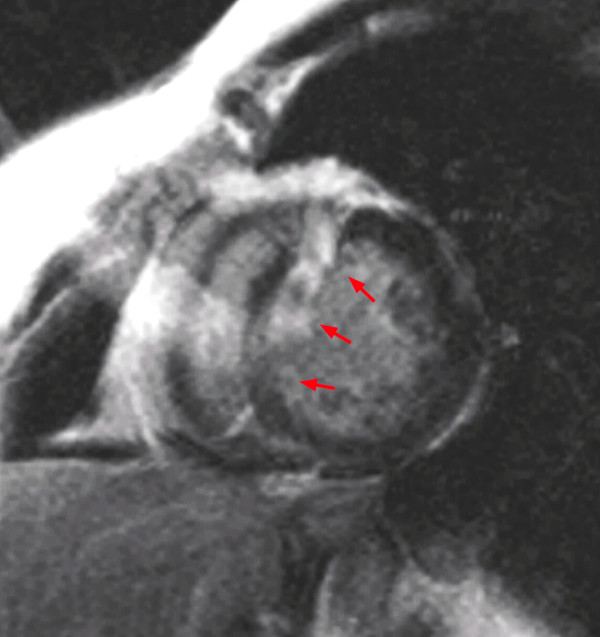
Wrap around artifact in a LGE sequence: A structure outside the field of view is projected onto the LV (red arrows).

**Figure 9:**
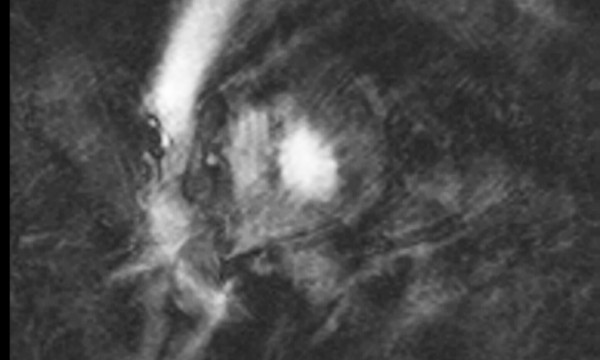
**ECG mis-triggering in a LGE sequence: Image quality is decreased by both, a mis-triggering artifact and by cardiac ghosts.** According the definitions of artifact criteria, the most severe artifact, i.e. the mis-triggering artifact, is considered for scoring only.

In addition to these qualitative criteria, LGE images were also evaluated by measuring 5 quantitative parameters including SI of the myocardium (anterior and inferior LV wall), the LV cavity, LV scar (if present), and noise. These quantitative measures were obtained by manually tracing a region of interest (ROI) in the above mentioned target structures in a midventricular SA slice, defined as the slice in the center position of the stack of SA LGE images (in stacks with a paired number of slices the more apical slice was evaluated, i.e. slice 6 in a stack of 10 SA slices with slice 1 at the base of the heart). The SI of the scar tissue was quantified only if a scar with >50% transmurality was observed. On the other hand, the myocardial SI was measured only if there was no scar in the segment of interest. Noise SI was measured in the air outside the patient. Finally the ratio between the myocardial SI and noise SI (SNR; criterion 36 in Figure 1) was calculated and scores were defined as follows: SNR <2 = score 0, SNR 2–5 = score 1, SNR >5 = score 2. These SNR measures to assess the correctness of signal nulling of normal healthy myocardium was chosen as the impact of varying contrast-to-noise thresholds (i.e. the ratio of scar signal vs remote healthy myocardial signal) is known to impact on infarct detection and quantification [13]. In cases with different scores in the anterior and inferior walls, scores of 0.5 or 1.5 were obtained through averaging. Notably, the LGE assessment was based on non-phase sensitive inversion recovery (IR) images (= the magnitude images of IR acquisitions). If present, segmented inversion-recovery acquisitions were considered. If no segmented inversion-recovery images were available, single-shot acquisitions were analyzed. Additional examples of image artifact of LGE images are provided in Figures 8 and 9.

### First-pass perfusion CMR images

The analysis of the perfusion images was based on similar qualitative criteria as those described above (criteria 23–29). For the analysis of stress perfusion CMR images the first set of perfusion images was analyzed exclusively to guarantee that image quality of *first*-pass acquisitions was considered. Subsequent contrast medium injections for perfusion studies were not analyzed. Only non-corrected sequences were analyzed, i.e. sequences subjected to automatic motion-correction algorithms were not considered. Because perfusion CMR sequences are typically based on a single-shot acquisition, ECG mis-triggering and breathing motion lead to different artifacts as compared to cine- and LGE images, which are typically acquired with a segmented technique. As a consequence, specific criteria were defined for ECG mis-triggering and breathing-related artifacts. Breathing motion during the acquisition of the perfusion CMR images (criterion 33 of Figure 1) results in a drift or an abrupt displacement of the heart depending on the excursion of the diaphragm. ECG-trigger artifacts (criterion 34) lead to variable cardiac contours in subsequent images. In cases of severe mis-triggering, where no QRS complexes are detected the first-pass of contrast medium can be missed completely yielding a score of 3. Finally, atrial fibrillation (criterion 35), in-plane special resolution in the phase-encoding direction (criterion 24), duration of the acquisition window (criterion 25), and the adequate preparation of the patient (no intake of caffeine and anti-anginal drugs for 24 hours before CMR; criterion 26) were considered as well (for details, see Figure 1).

Additionally, quantitative parameters were obtained by manually tracing ROIs in a midventricular SA slice (i.e. the slice proximal to the level where papillary muscles are attached to the LV wall) at baseline and at peak SI during first-pass in both, the anterior and inferior LV walls. SI increase was calculated as percentage of pre-contrast baseline SI (criterion 37 of Figure 1) and scored as follows: <100% = score 2, 100%-200% = score 1, >200% = score 0. These categories of SI increase were chosen as they are known to impact on diagnostic accuracy [14]. In cases with different scores in the anterior and inferior walls, scores of 0.5 or 1.5 were obtained through averaging. Peak SI was measured only if no perfusion defect was present in the segment of interest.

### Measurement of the inter-observer variability

#### Learning Phase

As a first step, the study nurse and the medical student got a detailed and intensive training on CMR image artifacts by SCMR level 3 experts. The teaching was aimed at understanding the basics about the causes and the appearance of all image artifacts mentioned above. During this learning-phase, differences in the assessment of the images between the different observers helped to redefine criteria where needed to improve reproducibility. In a next step, the criteria described above were then applied in a test set of 20 patients scanned at 1.5T. These studies were jointly interpreted by 2 CMR expert cardiologists (SCMR level 3), 1 study nurse, and 1 medical student. The study nurse and the medical student compared their results with those of a SCMR level 3 expert every 5 patients in order to obtain a detailed feed-back on their assessment, to answer their questions regarding applications of criteria, to ensure that a complete CMR examination can be evaluated within 15–20 minutes, and to finally improve the accuracy of their analyses.

#### Validation phase

After completion of the learning phase, the two CMR experts, the medical student and the study nurse independently performed a quality assessment of another 30 patients (15 males and 15 females) scanned at our institution at 1.5T and another 30 patients scanned at 3T (15 males and 15 females). Additionally, 30 cases randomly chosen from the Euro-CMR registry were analyzed with the same criteria by all investigators. The anonymous Euro-CMR registry cases were sent from the center of Ludwigshafen to a local server at our institution using a secure connection (ReverseProxy i-Sentry from BEEWARE). The connection is based on HTTPS (http over SSL) for all traffic between the client and the server. All the data is encrypted (login and password, and all dicom packets, encryption algorithm is sha1RSA and the public key is an RSA 1024 bits).

### Measurement of the intra-observer variability

To assess the intra-observer variability, 1 CMR expert cardiologist and the medical student repeated the analysis of 15 cases scanned in our institution at 1.5T and 15 cases scanned at 3T. To avoid a bias, this second reading session was performed at least 1 month after the completion of the first reading session. Furthermore, these 30 cases were randomly selected and all readers were blinded for the identity of the patient.

### Statistics

For the qualitative variables the inter- and intra-observer variability was assessed as the mean score and the mean score difference±SD between pairs of readers [15]. In addition, for pairs of readers the correlation coefficient was calculated by linear regression analysis. For the quantitative variables derived from the LGE and perfusion images, the SNR and the SI increase during first pass, respectively, were scored as described in the methods section. Finally, the percentage of agreement between readers was calculated (the sum of all scores that did not differ between 2 readers is expressed as percentage of all paired scores). Agreement was calculated for 6 pairs of readers for all patients (n=90) as well as for the subgroups studied at 1.5T, 3T (at our own institution), and the Euro-CMR cases studied at 1.5T. The intra-class correlation coefficients were calculated for the global quality score as well as for the quality scores of the 3 sub-modules (cine SSFP, LGE, first-pass perfusion) applying a 2-way mixed effects model. In addition, Cohen’s kappa was calculated for the pairs of 4 readers to assess the agreement for category assignments. Analyses were performed using the commercially available statistical package (SPSS version 19.0, IBM).

## Results and discussion

**Figure 10:**
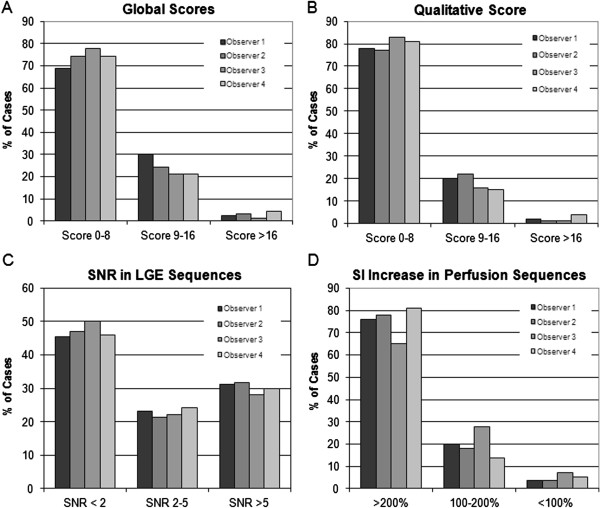
**Distribution of qualities in the entire study population (n=90) for the global quality score (A) as well as for the qualitative (B) and quantitative scores (C, D).** The first tertile of quality scores (score <9) encompasses the largest portion of studies ranging between 69% - 78% of all cases (for readers 1–4). A similar distribution is observed for the qualitative and the quantitative perfusion score, whereas the LGE score shows a considerable portion of increased signal in the normal myocardium of 21-24% (SNR 2–5) and 28-32% (SNR >5) of all studies indicating a sub-optimal myocardial signal nulling in these cases.

In the validation phase, by each observer a total of 3150 criteria (90 patients × 35 criteria) were assessed and 900 ROI (90 patients × 10 parameters) were measured. The global quality score, i.e. the combination of the total qualitative and the two quantitative scores of the entire study population (n=90, 1.5T, 3T, 1.5T of Euro-CMR registry cases) ranged from 0 to 24 for the 4 readers. When analyzing the spectrum of qualities in terms of tertiles (i.e. scores <9, 9–16, >16), 69% - 78% of all cases (for readers 1–4) fell into the first quality tertile (= best quality, scores <9, Figure 10A), and 21% - 30% and 1% - 4%, fell into the 2. tertile (scores 9–16) and 3. tertile (scores >16), respectively (Figure 10A).

In Figure 10B-D the break down into the qualitative and the 2 quantitative components of the global quality score is shown. The studies were well assigned to the 3 different tertiles by all 4 readers for all 3 sub-analyses (for the qualitative score, Figure 10B, as well as for the 2 quantitative scores, Figure 10C/D). The qualitative score (Figure 10B) yielded similar results for the 4 readers indicating that the qualitative criteria were well defined and reproducible.

The total qualitative score was 6.7±4.6 (n=90, mean of 4 observers) and ranged from 6.4-6.9 for the 4 observers (p=0.76 between observers). It ranged from 4.0-4.3 for 1.5T (p=0.96 between observers), from 5.9-6.9 (p=0.33 between observers) for 3T, and from 8.6-10.3 (p=0.40 between observers) for the Euro-CMR cases.

### Reproducibility of scores

For the global score the inter-observer agreement for the 6 possible comparisons ranged from 80% to 88%, see Table 1. The inter-observer agreement ranged from 83% to 92% for the qualitative analysis, and from 78-84% and 81-91% for the LGE quantitative analysis and the perfusion quantitative analysis, respectively (Table 1).

**Table 1:** Inter-observer agreement of all cases (n=90)

**Reader**	**1 vs 2**	**1 vs 3**	**1 vs 4**	**2 vs 3**	**2 vs 4**	**3 vs 4**
**Global quality score: Qualitative and quantitative quality assessment**						
**Inter-observer agreement**	80%	86%	88%	81%	83%	82%
**Total qualitative score**						
**Inter-observer agreement**	84%	89%	92%	84%	83%	90%
**Total quantitative LGE score**						
**Inter-observer agreement**	84%	82%	78%	81%	81%	82%
**Total quantitative perfusion score**						
**Inter-observer agreement**						
	88%	81%	91%	86%	90%	87%
**Intra-observer agreement (n=30)**						
**Reader**					**1 vs 1**	**4 vs 4**
**Global score: Qualitative and quantitative quality assessment**						
**Intra-observer agreement**					98%	90%

Readers 1 and 2 are SCMR level 3 experts; reader 3 is a trained study nurse, reader 4 is a trained medical student.

SI, signal intensity.

Global score was calculated as the sum of the qualitative score and the two quantitative scores (for SNR in LGE images and for first past SI increase in perfusion stress images).

Qualitative evaluation is classified into tertiles: scores <9 / 9-16 / >16.

LGE: Three scores of SNR: <2 / 2-5 / >5.

Perfusion: Three scores of SI increase during first pass: <100% / 100-200% / >200%.

Similarly, good agreements were observed for the 3 sub-populations (1.5T own institution, 3T own institution, 1.5T of the Euro-CMR registry) as given in Tables 2,3 and 4.

**Table 2:** Inter-observer agreement of the 1.5T cases at CHUV (n=30)

**Reader**	**1 vs. 2**	**1 vs. 3**	**1 vs. 4**	**2 vs. 3**	**2 vs. 4**	**3 vs. 4**
**Qualitative quality assessment**						
**Total quality score**						
MeanΔ±SD	0.2±1.6	0.3±1.6	0.0±1.5	0.1±0.9	−0.3±2.0	−0.4±2.0
Mean score	3.5	3.4	3.6	3.3	3.5	3.5
**Total quality score**						
Correlation coefficient	0.86	0.85	0.90	0.96	0.83	0.81
**Inter-observer agreement**	97%	97%	100%	100%	97%	97%
**Quantitative quality assessment: SI analyses**						
***LGE: Agreement for SNR classes***						
SI anterior wall	92%	89%	74%	93%	81%	74%
SI inferior wall	77%	81%	70%	85%	77%	74%
**Perfusion: Agreement for classes of SI increase during first pass**						
SI anterior wall	100%	96%	92%	96%	96%	88%
SI inferior wall	88%	76%	84%	83%	87%	79%
**Global Assessment**						
**Overall agreement**	97%	97%	100%	100%	97%	97%

Readers 1 and 2 are SCMR level 3 experts; reader 3 is a trained study nurse, reader 4 is a trained medical student.

SI, signal intensity.

All p-values of the linear regression analyses are <0.05.

Qualitative evaluation is classified into tertiles: scores <9 / 9-16 / >16.

LGE: Three scores of SNR: <2 / 2-5 / >5.

Perfusion: Three scores of SI increase during first pass: <100% / 100-200% / >200%.

**Table 3:** Inter-observer agreement of the 3T cases at CHUV (n=30)

**Reader**	**1 vs. 2**	**1 vs. 3**	**1 vs. 4**	**2 vs. 3**	**2 vs. 4**	**3 vs. 4**
**Qualitative quality assessment**						
**Total quality score**						
Mean Δ±SD	0.1±2.0	1.5±2.3	0.8±1.4	1.4±2.8	0.7±2.4	−0.7±2.1
Mean score	4.9	4.2	4.5	4.1	4.5	3.8
**Total quality score**						
Correlation coefficient	0.79	0.64	0.86	0.56	0.70	0.66
**Inter-observer agreement**	87%	93%	93%	80%	80%	100%
**Quantitative quality assessment: SI analysis**						
***LGE: Agreement for SNR classes***						
SI anterior wall	80%	81%	82%	74%	79%	88%
SI inferior wall	83%	81%	71%	81%	64%	77%
***Perfusion: Agreement for classes of SI increase during first pass***						
SI anterior wall	96%	86%	90%	89%	86%	82%
SI inferior wall	83%	70%	74%	83%	79%	67%
**Global assessment**						
**Overall agreement**	80%	87%	83%	80%	83%	83%

Readers 1 and 2 are SCMR level 3 experts; reader 3 is a trained study nurse, reader 4 is a trained medical student.

SI, signal intensity. All p-values of the linear regression analyses are <0.05.

Qualitative evaluation is classified into tertiles: scores <9 / 9-16 / >16.

LGE: Three scores of SNR: <2 / 2-5 / >5.

Perfusion: Three scores of SI increase during first pass: <100% / 100-200% / >200%.

**Table 4:** Inter-observer agreement of the Euro-CMR cases (n=30)

**Reader**	**1 vs. 2**	**1 vs. 3**	**1 vs. 4**	**2 vs. 3**	**2 vs. 4**	**3 vs. 4**
**Qualitative quality assessment**						
**Total quality score**						
Mean Δ±SD	0.9±3.6	1.9±2.9	−0.6±2.5	1.0±3.6	−1.6±3.6	−2.5±3.0
Mean score	8.4	7.9	9.2	7.5	8.7	8.2
**Total quality score**						
Correlation coefficient	0.76	0.82	0.89	0.74	0.78	0.85
Inter-observer agreement	70%	77%	83%	73%	73%	73%
**Quantitative quality assessment: SI analysis**						
***LGE: Agreement for SNR scores***						
SI anterior wall	90%	86%	76%	83%	86%	79%
SI inferior wall	83%	72%	79%	90%	83%	79%
***Perfusion: Agreement for scores of SI increase during first pass***						
SI anterior wall	76%	63%	80%	67%	90%	70%
SI inferior wall	81%	83%	73%	72%	79%	62%
**Global assessment**						
**Overall agreement**	63%	73%	80%	63%	70%	67%

Readers 1 and 2 are SCMR level 3 experts; reader 3 is a trained study nurse, reader 4 is a trained medical student.

SI, signal intensity.

All p-values of the linear regression analyses are <0.05.

Qualitative evaluation is classified into tertiles: scores <9 / 9-16 / >16.

LGE: Three scores of SNR: <2 / 2-5 / >5.

Perfusion: Three scores of SI increase during first pass: <100% / 100-200% / >200%.

**Figure 11:**
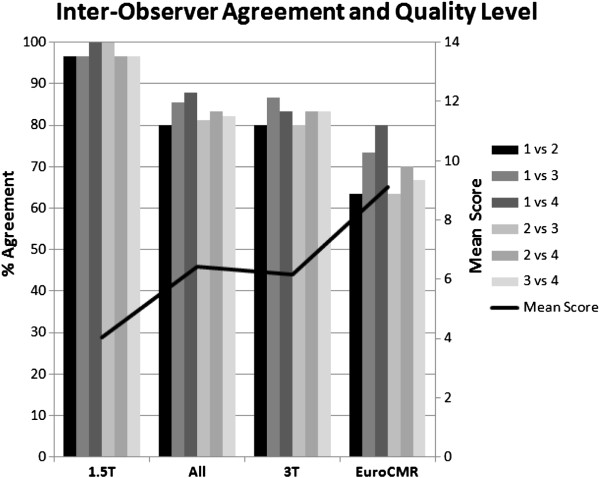
**Inter-observer agreement for the global quality score.** It ranges from 83% to 98% for the 4 readers (n=90 studies). As the level of quality deteriorates (ascending black line indicating mean global quality score of all 4 readers, units to the right of the figure), the level of agreement slightly declines, while for the good quality examinations at 1.5T (1.5T group to the left with a mean score of 4.0), the agreement between the 4 readers is excellent ranging from 97% to 100%.

For the studies performed at our own institution at 1.5T, reproducibility for the 4 observers was excellent with an agreement ranging from 97% - 100% for both, the qualitative and the global score (Table 2). The variability was slightly higher for studies performed at 3T at our own institution with an agreement for the qualitative score ranging from 80% – 100% and for the global score ranging from 80% – 87% (Table 3). Similarly, for the Euro-CMR cases the agreement ranged from 70% – 83% for the qualitative score and from 63-80% for the global score (Table 4). As illustrated in Figure 11 this higher variability was associated with higher absolute scores (= worse quality) in the group consisting of data acquired at different institutions and with different machines. It should be mentioned here, that this study was not designed to assess differences in quality between different scanners, different centers, or different field strengths, but to test the applicability and reproducibility of quality criteria to CMR data acquired during routine examinations. Therefore, no comparisons were made in regard to scanner type or field strengths.

The intra-observer variability was assessed for an experienced SCMR level 3 reader as well as for a trained medical student. As shown in Table 1, a good intra-observer agreement was obtained ranging from 90% to 98% for the global score of both readers.

**Figure 12:**
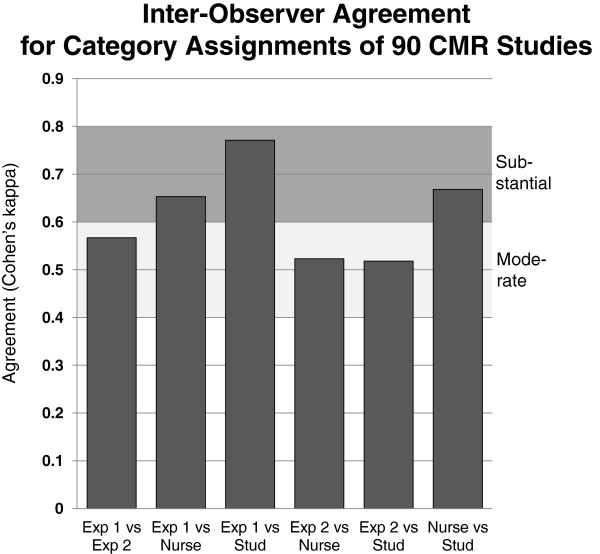
The various levels of agreement between experts (Exp), a trained study nurse (Nurse), and a trained medical student (Stud) is shown. For all comparisons, moderate to substantial agreements were found.

The intra-class correlation coefficient for the global quality score was 0.83 and for the quality scores of the sub-modules, i.e. cine SSFP, LGE, and first-pass perfusion images, coefficients were 0.83, 0.72, and 0.58, respectively. The Cohen’s kappa to assess agreement of category assignments by the 4 observers is given in Figure 12.

### Performance of the qualitative and quantitative quality criteria

This study describes the applicability and reproducibility of well defined quality criteria to evaluate cine SSFP, LGE, and first-pass perfusion CMR images, which are the most frequently used acquisitions in CMR. While the qualitative criteria are subjective to some degree, a strict definition was provided for each criterion with the final aim to enhance objectivity and reproducibility. The results of the current study show a good reproducibility between the quality assessments of multiple readers. This also holds true for examinations performed at 1.5T and 3T scanners, and for examinations performed in different institutions. To test the applicability of the quality criteria to data obtained at different field strengths and at different institutions was important to assess the reproducibility of the quality assessment. This study shows that the inter-observer reproducibility (expressed as percentage of the analysis classified in the same quality tertile) was acceptable ranging from 80% to 88% among the 4 readers (with an intra-class correlation coefficient of 0.83, see also Figure 12). The intra-observer reproducibility was excellent with values for global score agreement ranging from 90% to 98%. Notably, the inter-observer and intra-observer reproducibility was good not only for the comparison between CMR experts (i.e. cardiology experts in CMR), but also for the comparison with a well trained medical student. In summary, these favorable comparisons underscore the robustness of the described quality criteria.

While the total quantitative score ranges from 0 to a maximum of 4 only, it remains a relevant contributor to the quality assessment considering that the mean global quality score was as low as 6.7 points in the 90 CMR examinations evaluated.

### Applications of the quality criteria

These criteria will find their first application in the assessment of the image quality of the CMR examinations performed within the Euro-CMR registry [7]. Applications of these criteria will be of particular importance when used to evaluate the prospective sub-study examinations (protocols of suspected coronary artery disease, cardiomyopathies, and heart failure) [7].

In addition, these quality criteria may serve as a tool to improve the assessment of CMR image quality. By means of a common set of criteria as suggested in this study, qualities between different CMR examinations should be easier to compare. Nowadays, the performance of pulse sequences is typically characterized by reporting SNR and contrast-to-noise ratio (CNR), while other aspects such as susceptibility to artifacts are often neglected. With the proposed criteria, which are heavily based on artifact assessment, this aspect is incorporated into the quality analysis, while reproducibility in our view still remains acceptable. Novel CMR pulse sequences could be evaluated in the future based on the traditional parameters such as SNR and CNR while the proposed artifact-based assessment could be added to provide a broader assessment of quality. A quality assessment based on the proposed criteria could also allow for comparisons of image qualities in different CMR studies.

Finally, these standardized quality criteria may be useful for an institution to evaluate their own CMR quality. The principle of artifacts described in the criteria may also be used for teaching of physicians and MR technicians interested in CMR. In addition this framework for quality assessment could be used for accreditation purposes of CMR centers or for achieving core lab status. It could also serve to ensure consistency among operators and over time in the same institution or to detect quality drifts with newer sequences.

### Limitations

The present study was not designed to compare the image quality of studies performed at 1.5T vs 3T. The 1.5T and 3T data were used to test the applicability of the quality criteria to artifacts related to the various field strengths. Also, the 3T data were acquired with a beta-version of a shimming procedure and thus, this image quality is not representative of an overall 3T image quality. Nevertheless, we would like to underline that it is advantageous to evaluate quality criteria in a data set with a relatively high level of artifacts. Scanners of the newest generations operating at 3T are equipped nowadays with advanced shimming protocols and image quality is expected to be improved in comparison to the results presented here.

In this study, only the most often used sequences, i.e. cine SSFP images, LGE images, and first-pass perfusion-CMR images were evaluated, other sequences like black-blood imaging, flow or angiographic acquisitions were not analyzed. It is planned to establish quality criteria for these acquisitions in a future work. Also, patient-related factors such as weight, arrhythmia, breath-holding ability were not assessed in this scoring system as they would directly affect image quality.

This study evaluated the applicability and reproducibility of quality criteria to different sets of CMR examinations. Future studies are needed to evaluate at which levels of quality, correct diagnoses and appropriate outcome predictions can be obtained.

## Conclusions

The described criteria for the assessment of CMR image quality are robust and yield an acceptable inter-observer reproducibility. Further research is needed to define the impact of the image quality on the diagnostic and prognostic yield of CMR examinations.

## Abbreviations

CMR: Cardiovascular magnetic resonance; FOV: Field-of-view; LGE: Late gadolinium enhancement; LV: Left ventricle; ROI: Region of interest; SA: Short axis; SI: Signal intensity; SNR: Signal to noise ratio.

## Competing interests

The authors declared that they have no competing interest.

## Authors’ contributions

VK SM and JS designed the study, contributed to the data acquisition, analysis, and data interpretation. They drafted the manuscript. NL, DL, GV, PM and CL contributed to the data acquisition, analysis, interpretation of results, and revised critically the manuscript. DN, GP, ML, AC vR, AW, OB and HM contributed to data collection, data interpretation, and made a critical revision of the manuscript. All authors read and approved the final manuscript.

## Authors’ information

S. Muzzarelli and V. Klinke share first authorship of this paper.
